# The Vessels Concerned in the Production of Phlegmasia Dolens

**Published:** 1864-12

**Authors:** 


### *The Vessels Concerned in the Production of Phlegmasia Dolens.*

Before the Obstetrical Society of London, Dr. Tilbury Fox read a paper upon this subject. The author first referred to Dr. Mackenzie's experiments as insufficient to determine the question of the production of phlegmasia dolens, and proceeded to argue that venous obstruction is followed by œdema only, that the action must be the same, whether the obstruction be produced locally or indirectly through a vitiated blood condition. If any difference in the two cases existed, the changes over and above œdema, which characterize the lesion as phlegmasia dolens, must be ascribed to the action of the blood state (which is absent in the locally produced disease) upon the general texture of the limbs. If this view be adopted, the influence of the veins is *nil*, and we must look for the explanation in a special action carried on between the blood and the tissues. That the clinical history forbids the acceptance of such a doctrine, inasmuch as the very conditions (viz., blood vitiation tending to produce "phlebitis") which are regarded as the cause of phlegmasia dolens, very frequently exist, and yet are very rarely followed by white leg—for example, in the various blood-poisonings unconnected with the parturient condition. That if produced under the circumstances mentioned, the disease ought not to be so frequently unilateral nor confined to the lower limbs. That the occurrence of white leg in cases of cancer, phthisis, pressure, &c., could not be explained hereby. That the death-rate of phlegmasia dolens forbid the same interpretation of the phenomena. That in the experiments of the injection of lactic acid into the blood by Dr. Mackenzie, there was no evidence to show that in the dogs operated upon, anything but œdema resulted. That the existence of phlebitis, except in the rarest features, is fallacious in cases of venous disease. Attention was then drawn to the distinction between the coincidences and the essentials of phlegmasia dolens, as in the case of puerperal fever complicated by the latter. For example, take away from the general total of such a case the proper puerperal symptoms, and the phlegmasia dolens remains in perfect integrity: *per contra*, take away the hot, white, tense, elastic swelling, and the puerperal fever remains in its entirety.—In the combination, however, the pathological changes normal to phlegmasia dolens may be modified by the tissue actions (abscess, &c.,) which are the consequences of the existence of a virus in the blood; in uncomplicated phlegmasia dolens, the tissues are passive, so to speak.

The succeeding remarks went to show that the theory propounded by White was correct with regard to the *nature*, though not as to the *cause*, of phlegmasia dolens; that in the natural condition, a large quantity of lymph travels from the limbs towards the thoracic duct, and when this current is impeded, markedly white leg resulted. The case of the absorption of a poison into the cellular tissue (which, according to some, controverts White's opinion) was examined, and it appeared that this might or might not be followed by phlegmasia dolens, according as the obstruction in the symptoms affected the main current, or merely some minor channels, (the latter being the rule); the swelling being modified in severe cases, as before observed, by the relative action of the septic blood state and tissues. Cases were quoted to prove that lymphatic obstruction is sufficient, and alone necessary, to give rise to phlegmasia dolens. The paper concluded with the following summary:

1. Phlegmasia dolens is a local disease.

2. No general symptoms need be present (implying absence of blood-poison).

3. Phlebitis, however produced, cannot give rise to phlegmasia dolens, but œdema only.

4. Phlegmasia dolens may occur in, but forms no necessary part of, blood-poisoning, (such as tends to phlebitis), but is modified thereby frequently; and any tissue conditions over and beyond the presence of fibrinous serosity, and the consequent hypertrophous state of the areolar tissue, are in nowise essential components of phlegmasia dolens, but common alike to very many different "blood" diseases.

5. Obstruction to the main lymphatic channels alone is capable of giving rise to white leg, and acts by preventing the removal of the lymph from the affected limb.

6. The obstruction may be the result of (*a*) extrinsic pressure; (*b*) thrombosis due to sudden (compensatory) absorption of vitiated fluid after sudden loss of any kind; (*c*) inflammatory changes in the vessels themselves (rare).

7. The effect of the action of venous obstruction upon the phlegmasia dolens is an intensification of the general swelling, and the presence of œdema during the subsidence of the enlargement of the limb.

Lastly, a frequent but unrecognized source of blood-vitiation was alluded to, namely—in cases were large tracts of cellular tissue were diseased—as in erysipelas, sloughing, cancerous, phthisical and dysenteric ulcerations and the like—the lymphatics charged with effete matter, and an excessive number of imperfectly developed pale cells, formed in their glandular part, poured their contents into the venous system from the thoracic duct; and this might be a cause of thrombasis at the right side of the heart and in the vessels leading to the lung.

The author wished to know how phlegmasia dolens resulted in cases where no trace whatever of venous disease existed post mortem? and how it was, supposing blood-vitiation and venous obstruction to be the cause, that sometimes œdema and sometimes phlegmasia dolens resulted? why the swelling commenced sometimes above and sometimes below? why phlegmasia dolens, which was a greater degree of disease than œdema, resulting from blood-vitiation, (according to prevailing theories), possessed so low a mortality? It was evident in blood-disease, as appears from recent pathological inquiry, (especially brought out in the late discussion on puerperal fever at the Parisian Academy), that the lymphatics are as frequently affected as the veins, as in the broad ligaments of the uterus. A distinction must be drawn between thrombasis and inflammation of the lymphatics, which was usually due to septic poison, obstruction being uncommon, and certain tissue actions set going in answer to the blood state, modifying the whole character of any swelling. He did not throw cold water upon pathological facts, for the paper was based upon such; but thought that experimental evidence, such as that brought forward by Dr. Mackenzie, should not set aside clinical facts.

